# Diversity of current ultrasound practice within and outside radiology departments with a vision for 20 years into the future: a position paper of the ESR ultrasound subcommittee

**DOI:** 10.1186/s13244-023-01548-w

**Published:** 2023-11-24

**Authors:** Paul S. Sidhu, Caroline Ewertsen, Maciej Piskunowicz, Mustafa Secil, Paolo Ricci, Thomas Fischer, Diana Gaitini, Vladimir Mitkov, Adrian K. P. Lim, Qiang Lu, Wui K. Chong, Dirk Andre Clevert

**Affiliations:** 1 Department of Imaging Sciences, School of Biomedical Engineering and Imaging Sciences, Faculty of Life Sciences and Medicine, King’s College London, London, SE17EH UK; 2 Department of Radiology, King’s College Hospital NHS Foundation Trust, Denmark Hill, London, SE5 9RS UK; 3 Department of Radiology, Copenhagen University Hospital, Rigshospitalet, Copenhagen OE, Denmark; 4 Radiology Department, Medical University of Gdansk, Gdansk, Poland; 5 Department of Radiology, Faculty of Medicine, Dokuz Eylul University, Izmir, Türkiye; 6 Unit of Emergency Radiology, Department of Radiological, Oncological and Pathological Sciences, Sapienza University of Rome, Rome, Italy; 7 Department of Radiology, Interdisciplinary Ultrasound Center, Campus Charité Mitte, Charité, Universitätsmedizin Berlin, Freie Universität Berlin, Humboldt-Universität zu Berlin, and Berlin Institute of Health, Berlin, Germany; 8 Department of Radiology, Unit of Ultrasound, Rambam Medical Center and School of Medicine, Technion, Israel Institute of Technology, Haifa, Israel; 9 Diagnostic Ultrasound Department, Russian Medical Academy of Continuous Professional Education, Moscow, Russia; 10 Department of Imaging, Imperial College Healthcare NHS Trust & Department of digestive diseases, reproduction and metabolism, Imperial College London, London, UK; 11 Department of Ultrasound, West China Hospital of Sichuan University, Wuhou District Chengdu, China; 12 The University of Texas MD Anderson Cancer Center, Department of Abdominal Radiology, Division of Diagnostic Radiology, Houston, Texas USA; 13 Department of Radiology, University of Munich, Munich, Germany

**Keywords:** Ultrasound, Radiology, Perspective, International

## Abstract

Ultrasound practice is a longstanding tradition for radiology departments, being part of the family of imaging techniques. Ultrasound is widely practiced by non-radiologists but becoming less popular within radiology. The position of ultrasound in radiology is reviewed, and a possible long-term solution to manage radiologist expectations is proposed. An international group of experts in the practice of ultrasound was invited to describe the current organisation of ultrasound within the radiology departments in their own countries and comment on the interaction with non-radiologists and training arrangements. Issues related to regulation, non-medical practitioners, and training principles are detailed. A consensus view was sought from the experts regarding the position of ultrasound within radiology, with the vision of the best scenario for the continuing dominance of radiologists practising ultrasound. Comments were collated from nine different countries. Variable levels of training, practice, and interaction with non-radiologist were reported, with some countries relying on non-physician input to manage the service. All experts recognised there was a diminished desire to practice ultrasound by radiologists. Models varied from practising solely ultrasound and no other imaging techniques to radiology departments being central to the practice of ultrasound by radiologists and non-radiologist, housed within radiology. The consensus view was that the model favoured in select hospitals in Germany would be the most likely setup for ultrasound radiologist to develop and maintain practice. The vision for 20 years hence is for a central ultrasound section within radiology, headed by a trained expert radiologist, with non-radiologist using the facilities.

**Critical relevance statement** The future of ultrasound within the radiology department should encompass all ultrasound users, with radiologists expert in ultrasound, managing the ultrasound section within the radiology department. The current radiology trainees must learn of the importance of ultrasound as a component of the ‘holistic’ imaging of the patient.

**Key points:** 1. Ultrasound imaging within radiology departments precedes the introduction of CT and MR imaging and was first used over 50 years ago.

2. Non-radiology practitioners deploy ultrasound examinations to either ‘problem solve’ or perform a comprehensive ultrasound examination; radiologists provide comprehensive examinations or use ultrasound to direct interventional procedures.

3. Radiology does not ‘own’ ultrasound, but radiologists are best placed to offer a comprehensive patient-focused imaging assessment.

4. A vision of the future of ultrasound within the radiology department is encompassing all ultrasound users under radiologists who are experts in ultrasound, positioned within the radiology department.

5. The current radiology trainee must be aware of the importance of ultrasound as a component of the ‘holistic’ imaging of the patient.

## Introduction

In 2009, a commentary in European Radiology, written by two eminent radiologists with an interest in the practice of ultrasound within the radiology community, lamented the lack of interest and consideration given to the modality of ultrasound by radiologists [1], echoed by a similar view from North America [2]. This opinion had incredible insight into the ‘status’ of ultrasound within radiology, with predictions of the loss of this ‘artform’ of imaging from the radiologist’s tool box to other practitioners of ultrasound, lamenting the eventual loss of ultrasound to other medical specialities.

Twelve years later, radiology still has an ultrasound practice. The primary concern in 2009 was with ‘turf-battles’; but you can only have a ‘turf-battle’ if one party wishes to indulge in the continuing use of the modality; this is not the case with the Radiologist. An enormous amount of ultrasound is now performed by non-radiologists, but a substantial amount of ultrasound is still performed in radiology departments [3]. There is an increasing demand for imaging with ultrasound across all medical specialities, and globally ultrasound is the most used and pragmatic imaging modality, reflected by ultrasound machine sales. The advent of pocket ultrasound machines and smart telephones and tablets able to perform ultrasound increases the penetration of the modality into medical practice [4–6].

Radiologists continue to ‘tip-toe’ around the modality of ultrasound; trainees tend to favour the learning of computed tomography (CT) and magnetic resonance (MR) imaging before ultrasound, seeing the acquisition of ultrasound skills necessary for training but not needed when practising in the ‘real-world’ of radiology [7]. Often, ultrasound skills are only applied to a sub-speciality for example when used in musculoskeletal, breast, neck, or paediatric imaging. In fact, ultrasound is often perceived as a ‘chore’ by many radiologists but, paradoxically, is passionately embraced by non-radiologists, hence the explosion of use in the point-of-care scenario [8]. Advances and improvement in imaging technology derives from research, and the paucity of radiologists committed to ultrasound research is perturbing despite the outstanding improvements in ultrasound technology and the introduction of many ‘game-changing’ techniques (e.g. contrast-enhanced ultrasound, tissue elastography and fat quantification) [9–12].

Radiologists were conversant of the potential danger of losing ultrasound in 2009, and although ultrasound sections within radiology still exist, we are witnessing an explosion of non-radiologists using the technique. This situation will continue to evolve with radiology departments becoming less influential, likely detrimental to the overall imaging potential of radiologists’ practice. The purpose of this manuscript is to explore the current situation of the practice of ultrasound within the radiology community and document different practices within radiology departments across Europe and other parts of the world. An understanding of the direction for the radiological practice of ultrasound will be discussed with the concept of the future of ultrasound within radiology departments in 20 years suggested. The aim is to encourage and develop a sustainable ultrasound practice for radiologists.

## History of ultrasound in radiology

An understanding of the role of the radiologist in the development of ultrasound imaging is important to have an impression of the current status of the modality within radiology and the possible direction.

The progression of development of ultrasound was consequential on efforts of physicists, military technologist, engineers, and physicians of various disciplines. Initially, ultrasound was deployed in medicine in the visualisation of the brain in 1942 by an Austrian neurologist, Karl Dussik [13, 14]. In 1948, George D. Ludwig, an internist at the Naval Medical Research Institute, developed A-mode ultrasound equipment and detected gallstones. Following this, John J. Wild, a surgeon, acquired the first B-mode images [15–17]. The first radiologist to ‘dabble’ with this new imaging modality was Douglas Howry, who in 1952 focused on ultrasound technology and created a team to develop diagnostic quality ultrasound images by 1955 [18, 19]. Subsequently, ultrasound proved to be a clinically usable diagnostic tool supported with practical experience and scientific research, which eventually directed the technology manufacturers to develop and produce ultrasound devices between 1960 and 1970 [20]. This preceded the introduction of CT and MR imaging techniques. Ultrasound initially gained popularity outside radiology particularly in obstetrics, gynaecology, and cardiology.

In 1993, a radiologist, Barry Goldberg, first described the many challenges confronting ultrasound as an imaging discipline of radiology, tracing conflicts with other physicians dating from the 1960s [21]. A practical issue with ultrasound for radiologists, more experienced in the use of X-rays, was the perceived inherent limitations of ultrasound, difference in tissue representation, relatively low resolution, and difficulty in imaging tissues such as lung, bowel, and bone. Consequently, minimal departmental support was provided for ultrasound in radiology units, e.g. at the Royal Marsden Hospital in London; ultrasound was placed in the Nuclear Medicine Department. In the late 1970s and mid-1980s, CT and MR imaging were introduced into radiology departments. Although these new imaging techniques introduced additional workload and necessitated learning new concepts, these new methods were relatively more familiar and physically more comfortable diagnostic tools for the radiologists compared to ultrasound. By the 1980s, radiologists were performing about a one quarter of recorded ultrasound procedures in US hospitals [22].

Over the subsequent years, with improvement in the power of computing, Doppler ultrasound, power Doppler ultrasound, and 3D and 4D technologies were implemented in the ultrasound device [14]. Initially, radiologists were comparatively familiar with the developments, and adaptation was embraced with ease.

Further developments occurred but with less enthusiastic adaptation by radiologists; ultrasound contrast agents, tissue and liver elastography, were introduced to daily practice. These newer techniques have very rapidly become familiar to non-radiologists, adapted with enthusiasm, predominantly a consequence of not having ready access to CT and MR imaging. Unlike CT using ionising radiation, no legal constraints exist for ultrasound, which is also relatively inexpensive in comparison to CT and MR equipment. A conclusion from the epilogue of Barry Goldberg’s 1993 article—despite the atmosphere of resistance, radiologists persevered and made progress in the development of diagnostic ultrasound’ [21]—may no longer be the ethos of the younger radiologist, with innovative techniques being less familiar.

## How many radiologists include ultrasound in their practice?

The number of radiologists who continue to use ultrasound in their daily practice was recently documented in a survey conducted by the European Society of Radiology [23]. Respondents were asked to describe the distribution of modalities that they use in their own clinical practice. The top three modalities practiced by respondents were CT (high or very high use in 73%), MR (high or very high use in 64%), and ultrasound (high or very high use in 55%). In effect, a little more than half of attending radiologists continue to use ultrasound in their radiological practice, less than plain films; 88.5% continued reporting plain films in their practice, outstripping CT and MR reporting. There does remain a cohort of radiologists dedicated to a continuing ultrasound practice, and this should be recognised, supported, and encouraged.

The day-to-day running of a general ultrasound section is different in various countries, and dedication to performing ultrasound may fall to a few individuals in each department, with predominantly specialist ultrasound in areas such as breast, paediatrics, and musculoskeletal practice predominating. The task of ‘on-call’ ultrasound will normally be the responsibility of the radiology trainee or not offered at all. The probability will be that a proportion of radiologists do maintain some ultrasound in their practice, but it is likely to be a small proportion; some radiologists will not include any ultrasound in their imaging practice [23].

## Training the ultrasonographer: education in Europe

Trainee radiologists will have ultrasound involvement as a core requisite in the curriculum and are instructed by the radiology trainers, by definition competent in ultrasound techniques and diagnosis. Any clinician, both radiologist and non-radiologist desiring to use ultrasound in their practice, should have the opportunity to be educated by experts. In other clinical skills, such as endoscopy and surgery, medical specialties have defined which training and tasks should be completed to fulfil different levels of competencies. This could also be applied to ultrasound (as is already applied in some countries, when medical subspecialties use ultrasound) with a basic technical education followed by different levels of skills within different anatomical areas. Education and training should be delivered by experts in the ultrasound field—in radiology, this is normally provided by trained radiologists and sometimes by trained ultrasound technicians or sonographers. Invariably radiology trainees are examined in competency prior to qualification.

### Ultrasound practice in different healthcare systems

The practice and the position of ultrasound in various healthcare systems across the world are variable, with the involvement of the radiology department based on the practice of local physicians, surgeons, and primary care doctors. The remit of training and dissemination of ultrasound knowledge may also be quite variable in radiology departments, essentially dependent on the local hospital requirements and the position of ultrasound services in the national healthcare systems. A snapshot, based on the personal experience of the authors, of the remit of ultrasound sections in hospitals across Europe, China, and the USA gives insight into challenges facing the practice of ultrasound within radiology.

### Germany

Guidelines for medical ultrasound examinations in Germany are primarily provided in the regulations for residency training and ultrasound agreements for the different specialties. In Germany, organ-related ultrasound examinations are performed by radiologists as well as clinicians from the respective specialties under the guidance of the German Society for Ultrasound in Medicine (Deutsche Gesellschaft für Ultraschall in der Medizin, DEGUM). All radiologists in Germany should receive training in ultrasound, lasting between 3 and 6 months, and require certification confirmation from the department head on completion, in order to practice. Radiologists and other physicians performing ultrasound examinations have to keep pace with ongoing technical developments and ensure dedicated and time-consuming training of junior physicians. Provision of staff-intensive on-call services for ultrasound examinations may pose a challenge, especially for small departments. As ultrasound equipment requires regular maintenance and is costly to replace, some departments work with outdated equipment. Reimbursement for ultrasound examinations performed by medical practitioners has been lowered, and many hospitals have a large number of ultrasound machines without making full use of them, which has led to a waste of resources in hospitals. Nevertheless, many specialties insist on continuing to run their own ultrasound sections in an attempt to defend their ‘turf’, also in order not to lose their authorisation to provide ultrasound residency training. A possible strategic way out of this situation could be interdisciplinary cooperation in an ultrasound centre. Model ultrasound centres exist in Munich, Rostock, Regensburg, and Berlin [24]. As with other cross-sectional imaging modalities, such ultrasound centres operate under the roof of radiology. Ultrasound is part of the radiological specialist training in Germany; in the night services, the trainee radiologists often perform the ultrasound examination and offers a comprehensive 24-h service.

### Italy

Radiology training course includes attendance in the ultrasound section in the first 3 years of training in radiology, with an expectation of 6–8 months in total. Second- and third-year residents personally carry out US examinations under the supervision of an expert tutor. Ultrasound activity is also carried out during the attendance in the interventional radiology sections. Presently, in Italy, ultrasound is performed exclusively by medical practitioners, without any involvement by non-medically qualified practitioners (sonographers). No uniform regulation or organisation for ultrasound exists in the National Health Care System, which is sub-organised into regions with some local autonomy. Radiologists regularly perform ultrasound as part of their daily work practice, and residents are taught by experts, with the expectation of using these skills in their subsequent practice. In other specialties, non-radiologists perform ultrasound, for instance in the emergency department, and some specialties also perform ultrasound both for outpatient and inpatients, as well as in private practice, still within the National Health Care System. The necessity for standardisation of ultrasound practice obliged two organisations, the Italian Society of Medical and Interventional Radiology (SIRM) and the Italian Society of Ultrasound in Medicine and Biology (SIUMB), to establish a task force group, which produced a document called ‘Sonographic Medical Act’ [25], in which the necessary practice, training, and skills for ultrasound practitioners were defined in detail. The two societies have the aim of regulating sonographic examinations, so that these examinations are performed only to complete a clinical assessment and to answer only specific clinical questions, as defined by the Sonography-Assisted Medical Examination (SAME) [26]. However, further mandatory regional and governmental regulations are needed to establish these regulations. Therefore, with strict regulations in place to ensure medical practitioners perform ultrasound, radiology departments are at the forefront of the practice of the modality, with comprehensive training in position.

### Turkey

In Turkey, radiologists play a pivotal role in the ultrasound service. Obstetrics, gynaecology, and cardiology departments practice ultrasound within their own sphere, documenting structured reports based on the diagnostic findings. However, other medical and surgical specialities completely rely on radiology departments for nearly all ultrasound examinations. Although some ultrasound is performed by non-radiologists, there is no appropriate clinical report or appropriate image recording by these practitioners. In many academic state hospitals and private practice, radiology is still the only specialty to conduct a comprehensive ultrasound examination with a formal standard report, documenting the identified pathology [5, 27]. Ultrasound-practising gastroenterologists, rheumatologists, and emergency physicians are increasingly willing to take the responsibility for documenting findings in a formal written report. However, only a few enthusiastic physicians, who are well trained, are willing to undertake systemic ultrasound examinations. In Turkey, there is no law to restrict the application of ultrasound by any physician in their medical practice; although when challenged, the law authorities have placed restrictions that regulate primary care physicians, barring primary care physician from issuing written reports from an ultrasound examination. The public service reimbursement rules dictate that if an ultrasound is performed and reported by a non-radiologist specialist, reimbursement is less than that which is normally reimbursed to a radiologist. Since reimbursement is only symbolic (2–10 euros), many physicians do not document a report and use ultrasound as a continuum of the physical examination. Ultrasound education is a major part of radiology residency and takes a minimum of 1 year, which constitutes almost one quarter of the overall curriculum. In obstetrics, gynaecology, and cardiology, ultrasound education is also an important part of the training programme. In all other specialties, ultrasound education of residents remains unstructured, essentially due to a lack of sufficient educators and teachers.

### Israel

The Israelian health care system is based on the National Health Insurance Law of 1995. All citizens are required to join one of four official health insurance organisations. Health care is provided mostly in public hospitals and external clinics, maintained by the national government and the health insurance organisations. Ultrasound units in hospitals and community health providers are part of the radiology department. Examinations are performed by sonographers, normally trained radiographers, and supervised by a radiologist [28]. In the last 10 years, point-of-care ultrasound (POCUS) examinations within the emergency department and clinical and surgical wards are increasingly performed by non-radiologists. Programmes for teaching and training non-radiologists in the performance of POCUS have been implemented, in the last few years, by radiologists specialised in ultrasound. Interventional procedures under ultrasound guidance are performed by radiologists. Advanced ultrasound techniques like contrast enhanced ultrasound, elastography, and 3D ultrasound as well as research are performed in the radiology departments, mainly in university hospitals. An increase in the practice of POCUS by clinicians is predicted with further development of radiology-based ultrasound units in the area of teaching and research and the application of advanced techniques with modern ultrasound equipment, for better ultrasound diagnoses and therapeutic procedures. Radiology residents training in ultrasound spend 6 months in the ultrasound unit, together with chest x-ray interpretation, before starting the on-call work. During the on-call shifts, they perform and supervise the ultrasound examinations, subsequently reviewed by the senior ultrasound radiologist. Ultrasound imaging is part of the written and oral board examinations.

### Denmark

In Denmark, all radiology departments offer ultrasound examinations and interventions, and it is part of the curriculum for the residents in radiology to be able to do ultrasound-guided intervention in the pleura, breast, and abdomen. Denmark has a long tradition for ultrasound and ultrasound-guided intervention. Except for abscesses in the breast, all ultrasound examinations of the breasts are performed by dedicated breast radiologists. Some anatomical areas are never examined within the radiological department, but by other medical specialties, which include training in their curricula. Gynaecologists and obstetricians perform all ultrasound examinations of the female reproductive organs and all foetal scans. Cardiologists perform echocardiography, and vascular surgeons perform ultrasound preoperatively. In recent years, some rheumatologists have included ultrasound in their examinations of joints, and some otorhinolaryngologists perform ultrasound of the neck. Other medical specialties include ultrasound as a point of care examination, for instance intraoperative ultrasound in liver surgery and prostate ultrasound by urologists. Emergency medicine doctors and general practitioners may use POCUS to confirm or rule out specific diagnoses as gallbladder calculi, hydronephrosis, aortic aneurysm, pneumothorax, and free intraabdominal fluid. Also, anaesthesiologists use ultrasound for vascular access and in the ambulances and helicopters transferring patients.

### Russia

Until 1984, Russia had a small number of modern ultrasound equipment mainly in research and educational medical institutions. Since 1984, modern ultrasound systems have begun to arrive in the country in large (for this period) quantities, estimated at > 400 systems per year. Only doctors were entrusted with conducting ultrasound examinations with these sophisticated and expensive ultrasound systems. By 1987, the need to organise a regular systematic training of doctors performing ultrasound examinations in the country became obvious. Two main factors were taken into account: the vast territory of the country with a large number of regions with an extremely low population density and the lack of ultrasound equipment and doctors capable of using this equipment. The solution was to concentrate the equipment in one place and training doctors, who would be employed full-time to do ultrasound examinations. In 1988, by order of the USSR (Union of Soviet Socialist Republics) Ministry of Health, interdisciplinary departments of diagnostic ultrasound were created, and the specialty of ultrasound diagnostics appeared. The advantages of interdisciplinary ultrasound department are as follows: the possibility of more efficient use of ultrasonic equipment (especially of a high level), reducing the waiting time for the study by the patient, a one-step study of various organs and systems in a patient, high specification of an ultrasound diagnostics doctor whose daily work improves the level of expertise and skill, easy redirection of the patients flow in case of illness of one of the doctors, affordable and quick consultation with colleagues. The ultrasound-based doctors are not radiologists; they are from different specialties (including radiology), but after additional training (from 500 h) and passing state accreditation (‘OSCE’), they are accredited in the specialty of ultrasound.

### United Kingdom

Diagnostic ultrasound in the UK has over the years devolved from radiologists to sonographers (usually radiographers who have gained postgraduate qualifications); it is estimated that 85% of all ultrasound scans in the UK are performed by sonographers [29]. Their training and skills are comprehensive and, in many areas including some obstetric, gynaecological, and general applications, may exceed those of a radiologist. Frequently, in the UK, hospitals have sonographer-led services including general, contrast enhanced ultrasound and paediatrics, and it is not uncommon for sonographers to provide training to radiologists [30]. Some specialised ultrasound examinations, for example, musculoskeletal, head and neck, breast, and interventional ultrasound, are still predominantly performed by radiologists, although more sonographers are now becoming skilled in these techniques as demand continues to escalate. The USA and Australia have similar skilled sonographers, but generally only those in the UK report their findings independently. There are now a small but increasing number of non-radiology clinicians performing focused ultrasound for a quick diagnosis to inform patient management including obstetricians, anaesthetists, intensivists, general medical and emergency department physicians, and allied health professionals such as physiotherapists. The landscape of ultrasound users in the UK is broadening as a consequence of the modality’s wide appeal and versatility. Radiology training has been part of the core curriculum within radiology and with this ultrasound training, the trainees are expected to deal with all aspects of ultrasound whilst performing their on-call duties.

### West China Hospitals

Ultrasound is the most widely used imaging modality in China. It is estimated that there are around 190,000 ultrasound machines nationwide [31] Moreover, the need for ultrasound examinations is still on the rise, and the number of ultrasound practitioners is inadequate. Unlike the ultrasound section incorporated into the radiology departments in most countries, the ultrasound department is an independent unit in China, very much similar to the situation in Russia. In addition, the majority of hospitals in China have only ultrasound physicians performing both the scanning and diagnosis and reporting of the examination. In order to meet the huge and diverse needs for ultrasound since 2007, West China Hospitals, a group of geographically adjacent hospitals in the Western part of China, has been trying a new working model in China by employing both ultrasound physicians and sonographers. The demand for ultrasound can be stratified into four levels: (i) POCUS, (ii) more basic common ultrasound examinations, (iii) more specialised ultrasound examinations, and (iv) interventional ultrasound. Staff with different experiences and educational backgrounds are appointed to meet the corresponding demands. Ultrasound physicians play a vital role in this working model with multiple responsibilities. Specifically, attending (consultant) and senior doctors are engaged in teaching, supervising common ultrasound examinations, and performing examinations for more complicated cases and ultrasound-guided interventional procedures. Sonographers are mainly involved in POCUS and common ultrasound scanning, examination description, and providing ultrasound examination ‘impression’, which is reported by the ultrasound physician. POCUS is becoming a part of the daily practice of some specialists, in particular emergency physicians, anaesthesiologist, and intensivist. Quality control is a key part of the working model. Besides multi-modular training, a real-time consultation platform integrating ultrasound information system (UIS) and audio–video system has been used to guarantee the quality of ultrasound service, especially those carried out by sonographers and junior ultrasound physicians. Moreover, for quality control, reports are scored weekly, including ultrasound images and text, which will be finalised by using artificial intelligence in the near future.

### United States of America

Ultrasound in the USA has traditionally been performed by the departments of radiology, obstetrics, and gynaecology and vascular surgery. Ultrasound examinations are usually performed by sonographers using structured protocols, with back-scanning and interpretation by physicians. Cine clips are widely used. Radiologists perform more scanning in musculoskeletal, paediatrics, and niche applications and during interventional procedures. Ultrasound is a required subject in radiology residency, but the amount of resident scanning is at the discretion of the programme. This traditional practice is being upended by advances in computing capability that have resulted in smaller and cheaper ultrasound devices. The handheld ultrasound scanner is a disruptive technology. It is poised to become the stethoscope of the twenty-first century, a ubiquitous tool used by all healthcare workers. This has given rise to POCUS in which ultrasound is performed as an adjunct to the clinical examination. POCUS is now practiced by a growing number of physicians and paramedical staff. Ultrasound scanning is now taught as a foundational skill in medical schools. As such, the practice of ultrasound will no longer be confined to trained specialists like radiologists, obstetricians, or sonographers. Most ultrasound scans in the USA now occur outside radiology departments. The future of ultrasound in radiology lies in focusing on quality, comprehensive examinations, and advanced modalities such as Doppler ultrasound, contrast-enhanced ultrasound, artificial intelligence, elastography, and 3D that require high-end equipment. Radiology resident scanning has been cut back, as all new trainees now start with ultrasound experience from medical school. The medical students are taught to scan during their emergency department and obstetrics and gynaecology rotations, like past generations were taught to use the stethoscope. All scanning protocols are based on practice parameters published by the American College of Radiology, American Institute of Ultrasound in Medicine, and Society of Radiologists in Ultrasound (ACR/AIUM/SRU), and these practice parameters act as a national quality standard of training, skills, techniques, and recommended conduct. There are over 30 ultrasound practice parameters (https://www.acr.org/Clinical-Resources/Practice-Parameters-and-Technical-Standards).

## Summary

The predominate European-based practice is for radiologists to be taught and learn the basic skills of ultrasound, during a training period, and is considered an essential aspect of radiological training. A similar training practice is largely encountered across European countries, with some minor differences. Usually, general aspects of ultrasound, particularly that involving the abdominal organs, are performed by radiology departments. The expectation that these examinations are covered by the radiology departments in the out-of-hours setting prevails and remains the duty of the radiology trainee.

Many subspeciality radiologists, e.g. musculoskeletal, continue the use of ultrasound in their daily practice; however, some others, e.g. gastrointestinal radiologists, may not. Many non-European countries, notably the English-speaking countries (e.g. Canada, USA, Australia), are sonographer dependent, whilst most European countries are predominantly physician based. Nearly all European countries have very strong non-radiologist practice of ultrasound, with ultrasound forming part of the curriculum for training, e.g. in urology and gastroenterology. Notwithstanding these differences, ultrasound generally remains a radiology tool, with established governance and structured training.

## Hypothetical direction of change for the practice of ultrasound

Directions of changes for ultrasound practice should take into account economic, human, and geographical factors. The growing financial outlay for healthcare in developed countries are related, on the one hand, to the salaries of the most qualified employees and, on the other hand, to the growing awareness and need for more preventive examinations for citizens of these countries. Thus, for maintaining cost-effectiveness and high availability of ultrasound examinations, the economic system naturally replaces the costly and highly specialised group of radiologists with mid-level personnel, in this case, sonographers [5, 7, 27]. This direction of change has been observed in wealthy countries with a large population, e.g. the USA and the UK. Moreover, these changes allow the transfer of radiologists to CT and MR imaging interpretation and reporting, perceived as requiring greater skill, where the number of examinations increases every year, with the inability currently to be replaced by mid-level personnel [32, 33]. However, in most European countries, ultrasound remains in the hands of physicians, and the sonographer’s programme is not available. This would appear to be partly related to cultural and legal considerations, though, in recent years, a trend has emerged in the young generation of radiologists who no longer feel threatened by non-physician sonographer practitioner [28, 34]. For the trainee radiologist, there is a noticeable decrease in interest in ultrasound, preferring training in MR and CT imaging techniques particularly with an emphasis on sub-specialisation in organ-based imaging in these modalities, not often offered in ultrasound.

One of the potential reasons for this change may be the decline in the ‘prestige’ in the view of the radiologist of the ultrasound examination. Currently, many more non-radiologist physicians perform ultrasound within the domain of their specialisation, although often only as a POCUS tool, but they do so with enthusiasm, knowledge, and skill. This increases the competitiveness on the ultrasound market and implies, in some healthcare systems which are fee based, lower remuneration for performing an ultrasound examination compared to an MR or CT imaging examination. Ultrasound is an essential component of paediatric, musculoskeletal, and breast imaging practice, the primary imaging modality for the foetus, thyroid, testis, and prostate, has a vital role in vascular disease, and is often the imaging guidance in interventional procedures. Outside of radiology, both cardiology and gastroenterology (in particular endoscopic ultrasound) view ultrasound as an essential imaging component. However, often, ultrasound is not considered part of the essential imaging ‘package’ for the patient in many areas of radiological practice, and ultrasound is usually delegated to a general pool of radiological imaging specialists—mostly radiology residents.

A timely question and discussion on whether radiology abandons ultrasound or rather if ultrasound abandons radiology is due, which is outside the scope of this article. However, if radiology abandons ultrasound, it will undoubtedly adversely affect the comprehensive framework of imaging methods available for patient care provided by the radiological community.

## Summary and conclusion

We have described the current practice of ultrasound in several European countries, USA, and China. Ultrasound is performed by different medical specialties and different health care personnel around the world. This varies among countries mainly depending on local tradition, but within recent years, this also reflects the increasing demand for ultrasound examinations. It has facilitated the performance of ultrasound examinations by other health care personnel, such as radiographers and nurses. In many countries, ultrasound was initially positioned under the umbrella of radiology, but it is important to bear in mind that initial research and practice was performed in a collaboration between engineers and different medical specialties, not just radiology, and has always been an inter-disciplinary modality. This is one of the reasons that ultrasound remains so attractive to practitioners from many medical specialties. The extraordinary diagnostic capability of ultrasound attracts many users particularly in the POCUS field, when immediate, safe, repeatable imaging allows for rapid diagnosis and appropriate patient care.

The future of ultrasound is not within the remit of the radiologist any longer, when once radiology departments reigned supreme in innovation and application of the modality, others have used ultrasound to manage patients in a timely manner. Where radiology still leads is in centralisation of comprehensive holistic ultrasound imaging, coupled to other techniques of imaging to best manage the needs of a patient, and importantly with well-established teaching and training in ultrasound to ensure a continuous high level of ultrasound practice. A possible strategic way out of this situation could be interdisciplinary cooperation in an ultrasound centre, as suggested in Germany [24], with entry into the specialty of ultrasound from various disciplines, not just radiology, and perhaps, ultimately using the West China Hospitals model, of support at various levels by trained sonographers.

## Data Availability

More information is available from the corresponding author on reasonable request.

## Abbreviations

ACR: American College of Radiology
AIUM: American Institute of Ultrasound in Medicine
CT: Computer tomography
DEGUM: Deutsche Gesellschaft für Ultraschall in der Medizin (German Society for Ultrasound in Medicine)
MR: Magnetic resonance
POCUS: Point-of-care ultrasound
SAME: Sonography-Assisted Medical Examination
SIRM: Italian Society of Medical and Interventional Radiology
SIUMB: Italian Society of Ultrasound in Medicine and Biology
SRU: Society of Radiologists in Ultrasound
UIS: Ultrasound information system
